# Genetic Polymorphisms in Base Excision Repair (BER) and Nucleotide Excision Repair (NER) Pathways as Potential Biomarkers for Gynecological Cancers: A Comprehensive Literature Review

**DOI:** 10.3390/cancers17132170

**Published:** 2025-06-27

**Authors:** Magdalena Szatkowska, Julita Zdrada-Nowak

**Affiliations:** Department of Cosmetology and Medical Biology, Wladyslaw Bieganski Collegium Medicum, Jan Dlugosz University in Czestochowa, 42-200 Czestochowa, Poland; j.zdrada-nowak@ujd.edu.pl

**Keywords:** biomarkers, gynecological cancer, SNPs, BER, NER, breast cancer, cervical cancer, endometrial cancer, ovarian cancer, personalized therapy

## Abstract

In 2022, approximately 1.4 million new cases of gynecological cancers were diagnosed worldwide, accounting for a significant share of all female cancer cases, according to the World Cancer Research Fund. DNA repair mechanisms play a critical role in maintaining genomic integrity, and their dysfunction can lead to the accumulation of DNA damage, thereby increasing the risk of gynecological cancer development. Single nucleotide polymorphisms (SNPs) in genes involved in DNA repair pathways, such as Base Excision Repair (BER) and Nucleotide Excision Repair (NER), represent important biomarkers for gynecological malignancies. These polymorphisms can affect the efficiency of DNA repair processes, thereby influencing individual susceptibility to cancer. SNPs within the BER and NER pathways exhibit high specificity, enabling accurate detection and monitoring of gynecological cancers, as well as the identification of individuals at elevated risk. This facilitates early risk assessment and supports the implementation of preventive strategies. Compared to traditional biomarkers such as CA-125, SNPs allow for the detection of genomic alterations at an earlier, preclinical stage. Furthermore, the characterization of SNPs in BER and NER pathways may serve as a foundation for personalized therapy, allowing treatment to be tailored to the patient’s specific genetic mutations. To identify polymorphisms in the BER and NER pathways associated with gynecological cancer risk, a systematic analysis of 128 scientific articles was conducted, which may serve as a solid foundation for advancing precision oncology and improving the early diagnosis of gynecological cancers.

## Simple Summary

Gynecological cancers, such as endometrial, cervical, and ovarian cancer, are among the leading causes of death in women worldwide. Scientists are searching for new ways to detect these diseases earlier and to tailor treatment options better. To achieve this, changes in genes responsible for DNA repair are being analyzed. When these repair processes are weakened, DNA damage can accumulate, increasing the risk of cancer development. In our study, we reviewed the latest scientific publications to identify genetic variations that may be linked to the development of gynecological cancers. These findings may contribute in the future to the early detection of these diseases and to the development of therapies tailored to the individual genetic profiles of patients.

## 1. Introduction

According to the World Health Organization (WHO), gynecological cancers, such as breast, endometrial, ovarian, and cervical cancers, are among the most frequently diagnosed cancers in women [1,2]. However, among the cancers listed, as many as 99% of cervical cancer cases are caused by infection with the human papillomavirus (HPV), particularly its high-risk oncogenic types, such as HPV 16 and 18, which are the most common [3]. In contrast, the etiology of other gynecological cancers, such as breast, endometrial, and ovarian cancer, is more complex and multifactorial. Risk factors include, among others, genetic predispositions (e.g., BRCA1 and BRCA2 gene mutations in breast and ovarian cancer), hormonal disorders, age, lifestyle, obesity, and environmental factors [4,5,6,7].

In 2022, approximately 46.1% of new cases of gynecological cancers resulted in death, highlighting the urgent need for more effective diagnostic and treatment methods. Given such high mortality rates, it is crucial to implement strategies that improve prevention and increase the availability of modern therapies to enhance survival rates and improve the quality of life for patients [1,2,8,9].

Genetic variability within the Base Excision Repair (BER) and Nucleotide Excision Repair (NER) pathways can significantly influence susceptibility to gynecological malignancies [10]. These pathways play a pivotal role in maintaining genomic stability by eliminating DNA lesions induced by genotoxic metabolites and reactive oxygen species (ROS) [11,12]. ROS, which arise from chronic inflammation, oxidative stress, and physiological processes such as menstruation, can cause a variety of DNA alterations, including base modifications, strand breaks, and other forms of genomic damage [13,14,15]. Single-nucleotide polymorphisms (SNPs) in genes encoding BER and NER proteins may impair the efficiency of DNA repair, thereby promoting the accumulation of mutations, particularly under conditions of elevated oxidative stress [16,17,18].

Gynecological tissues such as the endometrium, ovaries, and cervix are especially vulnerable to malignant transformation due to their hormonal responsiveness, high proliferative activity, and cyclic physiological remodeling [19,20]. Within this dynamic environment, even minor disruptions in DNA repair mechanisms can lead to the accumulation of genetic errors and initiate carcinogenesis [20]. Moreover, the BER and NER pathways contribute not only to tumor prevention but also to the regulation of tumor progression by modulating cell cycle control, apoptotic potential, and chromosomal stability [21,22]. Dysfunction in these pathways—often associated with deleterious SNP variants—can promote the emergence of aggressive tumor phenotypes, influence the tumor microenvironment through enhanced inflammation and angiogenesis, and contribute to resistance against anticancer therapies [23,24,25].

## 2. Causes of Gynecological Cancers

Gynecological cancers in women of reproductive age and in postmenopausal women result from multiple complex risk factors. These include, among others, the influence of hormones such as estrogens and DNA damage that may result from the activity of ROS [26,27]. In both life stages, hormones play a crucial role in cancer development, while uncontrolled ROS production and DNA damage constitute significant elements of the pathogenesis.

### 2.1. Menstruation and Inflammatory Processes Leading to DNA Damage

Numerous studies indicate that menstruation resembles a controlled inflammatory response that is essential for tissue regeneration. The shedding of the endometrium during menstruation is a process triggered by a rapid decline in estrogen and progesterone levels, leading to the activation of local inflammatory mediators [28,29,30,31]. This results in an increased concentration of pro-inflammatory cytokines such as interleukins 1, 6, and 8 (IL-1, IL-6, IL-8), as well as Tumor Necrosis Factor-alpha (TNF-α), within the endometrial tissue and ovaries [32]. In response to these pro-inflammatory stimuli, chemokines are produced, which amplify the inflammatory process by stimulating the recruitment and activation of neutrophils, macrophages, and other immune cells [32].

Neutrophils and macrophages play a key role in the activation of enzymes responsible for the production of reactive oxygen species (ROS), which act as signaling molecules that support the removal of sloughed endometrial cells and the regeneration of the uterine lining [33,34]. An increase in ROS production is a natural component of the inflammatory response; however, in the case of insufficient antioxidant defense systems, oxidative stress may develop, which has been associated with the onset of gynecological cancers [35,36].

A substantial body of evidence confirms that a deficiency in antioxidants—such as certain enzymes, vitamins A, C, and E, or glutathione (GSH)—exacerbates oxidative stress, increasing the risk of oxidative damage to DNA, lipids, and proteins. Under such conditions, chemotherapy often proves ineffective, complicating treatment and reducing its overall efficacy [37,38,39,40,41,42].

During oxidative stress, ROS interact with polyunsaturated fatty acids in cellular membranes, leading to lipid peroxidation and the formation of toxic by-products such as malondialdehyde (MDA) and 4-hydroxynonenal (4-HNE). MDA and 4-HNE play a dual role—they function as signaling molecules but also exhibit genotoxic properties. Their presence plays a critical role in the cellular response to oxidative stress and contributes to the pathogenesis of gynecological cancers [43,44].

**Figure 1:**
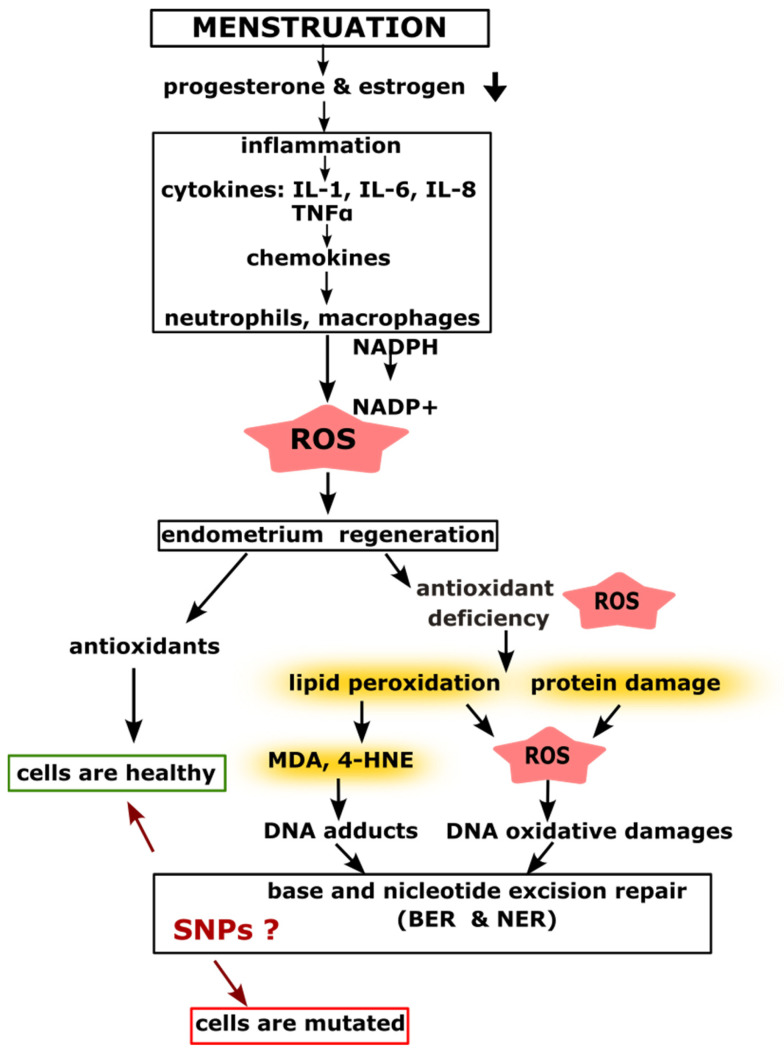
The diagram illustrates the role of SNPs in the BER and NER pathways during menstruation. A decrease in estrogen and progesterone levels triggers the activation of immune system cells, which supports regenerative processes in the endometrium, including tissue and blood vessel reconstruction. Antioxidant deficiency leads to lipid peroxidation in cell membranes and protein damage, initiating a self-perpetuating mechanism known as the oxidative stress cycle. DNA damage resulting from oxidative stress can be repaired through DNA repair pathways such as BER or NER. The efficiency of these repair processes is dependent on the polymorphic variants of proteins involved in the DNA repair systems.

ROS-induced modifications of nitrogenous bases in DNA may lead to mutations that increase the risk of malignant transformation. Lipid peroxidation products such as MDA can form adducts with deoxyguanosine (M1dG), which serve as substrates for DNA repair enzymes within the BER and NER pathways [45]. However, the efficiency of these repair mechanisms depends on the presence of polymorphic variants in genes encoding repair proteins. Impaired DNA repair capacity results in persistent oxidative stress, disrupting the cellular redox balance and further promoting carcinogenesis (Figure 1) [46,47].

### 2.2. Estrogen Dominance Leading to DNA Damage

Polymorphisms in genes involved in the BER and NER DNA repair pathways, combined with antioxidant deficiencies and excess estrogen, can significantly increase the risk of developing gynecological cancers [17,26,35,48]. Excess estrogen can stimulate cell proliferation in tissues sensitive to these hormones, such as the endometrial lining and breast tissue. Specifically, an imbalance between estrogen and progesterone, resulting in estrogen dominance, leads to tissue hyperplasia [49,50]. Chronic exposure to elevated estrogen levels can also induce a persistent inflammatory state in hormone-dependent tissues, which contributes to carcinogenic processes. Moreover, estrogen metabolites, such as 4-hydroxyestradiol (4-OHE2), exhibit pro-carcinogenic potential by causing DNA damage, particularly through the modification of guanine, leading to DNA adduct formation and oxidative damage [14,51].

After menopause, when ovarian estrogen production declines, estrogen levels may still increase in other tissues, such as adipose tissue, thereby prolonging overall estrogen exposure. Hormone replacement therapies—particularly those based solely on estrogen without the addition of progesterone—can elevate the risk of hormone-dependent cancers if not carefully tailored to the individual [52]. Furthermore, conditions such as polycystic ovary syndrome (PCOS), exposure to xenoestrogens, and excess adipose tissue, all of which contribute to elevated estrogen levels, may also increase the risk of developing hormone-dependent cancers [53,54,55].

Polymorphisms in BER and NER genes, which encode proteins involved in these DNA repair pathways, can affect the efficiency of repair processes, leading to a reduced ability to remove DNA damage, especially in the context of prolonged estrogen exposure. Such changes may promote the accumulation of genetic damage and consequently increase the risk of cancer development [56,57].

## 3. Methodology of the Systematic Review

**Figure 2:**
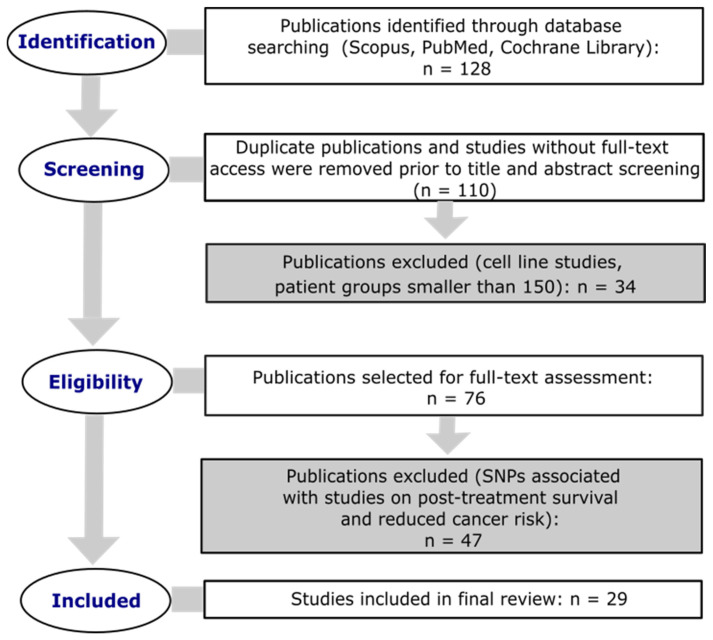
PRISMA search flowchart for the primary selected studies.

A literature review was conducted in accordance with PRISMA guidelines [58]. Database searches were performed using Scopus, PubMed, and the Cochrane Library to identify studies on SNPs in genes involved in BER and NER pathways that may be associated with an increased risk of developing gynecological cancers. A total of 128 scientific publications were analyzed as part of the review. Studies published between 2015 and 2025 were included. The search strategy was based on the following key phrases: “base excision repair” OR “nucleotide excision repair” AND “single nucleotide polymorphism” AND (“gynecological cancer” OR “cervical cancer” OR “endometrial cancer” OR “breast cancer” OR “ovarian cancer”). Only original research articles written in English were included in the analysis, provided they involved patient populations and control groups of at least 150 individuals each. Review articles and studies without full-text access were excluded from the review. The methodological framework employed in the present analysis is illustrated in Figure 2.

## 4. SNPs in BER and NER Pathway Genes in Diagnostics and Therapy

### SNPs Predict Susceptibility and Survival

Some polymorphisms in genes involved in DNA repair pathways, such as BER and NER, have been linked to an increased risk of gynecological cancers (Table 1 and Table 2). An example is the rs1130409 polymorphism in the APEX1 gene, which is associated with reduced DNA repair activity in the BER pathway. When combined with an increased ratio of estrogen-DNA adducts, this polymorphism may elevate the risk of breast cancer [59].

Furthermore, an increasing body of research points to the synergistic effect of SNP polymorphisms in the BER and NER pathways. For instance, the combination of the ERCC2 751Gln and XRCC1 194Trp variants shows a stronger effect on increasing the risk of endometrial cancer [75]. On the other hand, other studies suggest that the combination of the XRCC1 194Trp and 399Gln alleles is associated with a significantly reduced risk of ovarian cancer mortality, compared to the presence of these variants individually [76,77]. This effect highlights the role of these polymorphisms as both critical biomarkers for risk assessment and potential therapeutic targets.

The identification of SNPs in genes involved in DNA repair, such as those in the BER and NER pathways, is also crucial for predicting survival outcomes in gynecological cancer patients undergoing treatment. For example, certain studies have shown that women with breast cancer carrying the 399Gln/Gln genotype in the XRCC1 gene have better overall survival and progression-free survival outcomes when treated with adjuvant therapy, compared to individuals with other genotypes [78,79]. In response to platinum-based chemotherapy, ovarian cancer patients with the XRCC1 194Trp/Trp genotype had a longer survival time compared to patients with the Arg/Arg genotype. Additionally, patients with the XRCC1 399Gln/Gln genotype showed a 56% reduction in the risk of death compared to those with the Arg/Arg genotype [76].

**Table 2:** SNPs in DNA repair pathway genes—NER (data from 2015–2025).

Protein Name	db SNP ID	Number of Cases/Controls	Gynecological Cancer	References
XPA	rs1800975	2338/2731	breast cancer	[80]
	rs1805348	371/420	endometrial cancer	[81]
	rs2808667	783/795	endometrial cancer	[82]
XPC	rs2228000	400/400	cervical cancer	[62]
	rs2228001; rs2276466	210/200	cervical cancer	[83]
	rs2227998	300/300	breast cancer	[65]
	rs3731127	783/795	endometrial cancer	[82]
ERCC1	rs1799793	25,446/41,106	endometrial cancer	[73]
	rs3212986	9896/11,027	ovarian cancer	[84]
ERCC2	rs238406	400/400	ovarian cancer	[85]
		1360/1320	endometrial cancer	[86]
	rs1799793	400/400	cervical cancer	[62]
	rs13181	300/300	breast cancer	[65]
		610/610	endometrial cancer	[87]
		510/510	endometrial cancer	[63]
		1333/2691	ovarian cancer	[18]
		430/430	ovarian cancer	[88]
ERCC5	rs4150386	783/795	endometrial cancer	[82]
	rs17655	478/922	cervical cancer	[89]
	rs17655	4028/4953	cervical cancer	[16]
LIG1	rs3730865	783/795	endometrial cancer	[82]

## 5. SNPs in Personalized Therapy

Advances in research on genetic polymorphisms in DNA repair genes, such as those associated with the BER and NER pathways, contribute to the development of personalized therapies that account for individual genetic differences among patients [90,91]. The presence of specific variants, such as rs25487 in the XRCC1 gene and rs751402 in the ERCC5 (XPG) gene, can make cells more sensitive to the effects of oxidative stress-inducing drugs, such as doxorubicin, resveratrol, curcumin, or epigallocatechin gallate (EGCG) [92,93,94,95].

XRCC1 plays a crucial role in coordinating and stabilizing interactions between repair proteins, while the rs25487 polymorphism weakens these interactions, leading to decreased DNA repair efficiency in the BER pathway, particularly in ovarian cancer [96]. ERCC5 (XPG) encodes a protein that functions as a nuclease responsible for excising damaged DNA strands in the NER pathway. The rs751402 polymorphism in this gene may contribute to the development of breast cancer, primarily through a reduced ability to protect cells from oxidative stress [97]. Therefore, the coexistence of certain polymorphisms in DNA repair pathways may influence the efficacy of gynecological cancer treatments by increasing the sensitivity of cancer cells to chemotherapeutic agents [36,93,98,99]. Consequently, these polymorphisms form the basis for the optimization of personalized therapies, tailored to the individual genotype of the patient.

Polymorphisms in the BER and NER pathways can also significantly affect the effectiveness of gynecological cancer therapies using PARP (Poly (ADP-ribose) polymerase) inhibitors, such as olaparib, niraparib, or rucaparib [100,101]. PARP-1 plays a crucial role in the repair of single-strand DNA breaks (SSBs), which are primarily repaired through the BER pathway. Additionally, it has been shown that PARP-1 can also engage in the repair of SSBs that arise after the removal of damaged DNA sequences due to inefficient repair by the NER pathway [102,103,104].

Numerous authors have demonstrated that polymorphisms in genes encoding DNA glycosylases, such as UNG and POLG, can modify the risk of cancer development in carriers of BRCA1 and BRCA2 mutations, a high-risk group [105,106,107]. Similar correlations have been observed for polymorphisms in NER pathway genes, which have been associated with breast cancer risk and cervical cancer [108,109]. Incorporating analysis of these genetic variants into molecular studies may significantly improve the accuracy of risk assessment and contribute to the development of personalized prevention and treatment strategies for patients with BRCA mutations.

**Figure 3:**
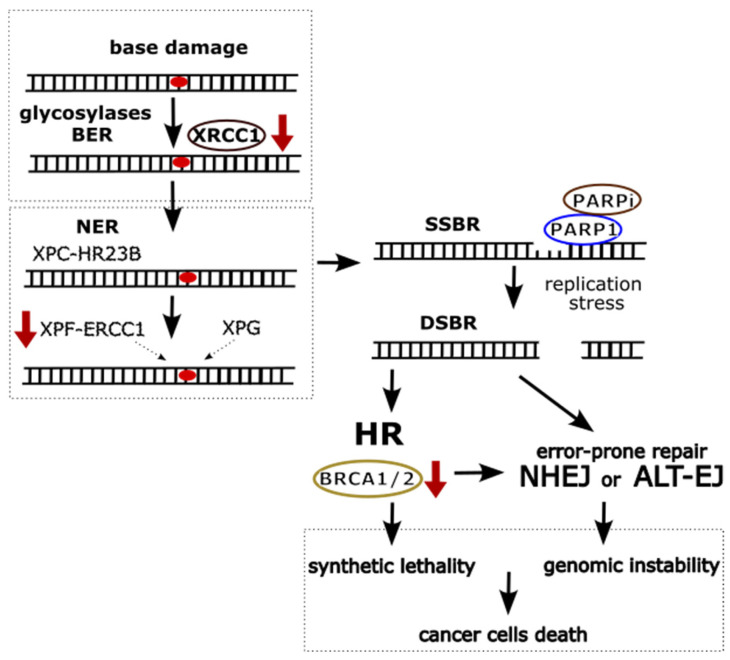
Interactions of DNA repair pathways: Base Excision Repair (BER), Nucleotide Excision Repair (NER), Single-Strand Break Repair (SSBR), Double-Strand Break Repair (DSBR), including Homologous Recombination (HR), Non-Homologous End Joining (NHEJ), and Alternative End Joining (ALT-EJ). The red downward arrow symbolizes reduced or absent activity of specific repair proteins, which may shift the balance of repair processes, forcing cells to rely on alternative, often less accurate pathways. This imbalance is particularly significant in the context of PARP inhibitor treatment and mutations in BRCA1/2 genes, where the loss of effective repair mechanisms leads to the accumulation of DNA damage, potentially resulting in synthetic lethality, in which cancer cells are selectively eliminated due to their inability to repair critical DNA damage.

Additional defects in DNA repair mechanisms, particularly in the NER pathway, can lead to increased reliance of cancer cells on alternative DNA repair pathways. Consequently, cells become more sensitive to PARP inhibitors, enabling the use of synthetic lethality strategies [110,111]. This mechanism involves the simultaneous blocking of two key DNA repair pathways—damage to one pathway leads to cell death if the other is also impaired [112]. Mutations in BRCA1/2 genes, especially in cancers such as ovarian, breast, and endometrial cancer, result in increased cellular sensitivity to PARP inhibitors [103,113]. Further impairments in DNA repair pathways such as BER and NER may exacerbate deficiencies in repair mechanisms, thereby enhancing the efficacy of PARP inhibitor-based therapies (Figure 3).

## 6. The Role of BER and NER Pathways in Tumor Regulation and Progression

As neoplastic transformation advances, cells become increasingly vulnerable to replicative stress, hypoxia, and oxidative damage. These conditions result in the accumulation of diverse DNA lesions [114,115,116]. In this context, the base excision repair (BER) and nucleotide excision repair (NER) pathways play a dual role: on the one hand, they safeguard genome integrity; on the other, they contribute to the survival of cancer cells within a hostile tumor microenvironment.

In gynecologic cancers, overexpression of BER-related proteins such as APE1 and XRCC1 has been associated with an aggressive phenotype, elevated genotoxic stress, and resistance to platinum-based chemotherapy [25,117,118]. The BER pathway is initiated by DNA glycosylases that recognize and remove damaged bases, triggering a repair cascade involving PARP-1 and activation of the ATR–CHK1–CDC25A/p53 axis. When DNA damage persists, it may lead to the formation of double-strand breaks (DSBs), which activate the ATM–CHK2–CDC25C pathway and result in G2/M cell cycle arrest, cellular senescence, or apoptosis [119].

The NER pathway, in turn, is responsible for removing bulky DNA lesions, such as chemical adducts and UV-induced pyrimidine dimers. This process begins with lesion recognition and the recruitment of the TFIIH complex, which contains XPB and XPD helicases that unwind the DNA around the damage site. This exposes single-stranded DNA and activates the ATR signaling pathway, ultimately leading to phase-specific activation of CDC25 [120,121]. Reduced expression of key NER genes, such as ERCC1 and XPA, has been associated with decreased sensitivity to cytotoxic therapies, underscoring the importance of NER in therapeutic response modulation [25,122].

Deficiencies in BER and NER function result in the accumulation of DNA damage, increased mutagenesis, and genomic instability—key drivers of tumor initiation and progression. Polymorphisms in genes involved in these pathways (e.g., XRCC1, APE1, XPD) have been widely investigated as risk factors for multiple cancer types, including gynecologic malignancies. Such genetic variability can influence DNA repair capacity and thereby affect susceptibility to mutational accumulation, cancer development, and tumor aggressiveness [17,59,123,124,125,126,127].

Beyond genome maintenance, the BER and NER pathways also modulate the tumor microenvironment and immune response. Efficient DNA repair in cancer cells reduces the mutational burden, thereby limiting the production and presentation of tumor-associated neoantigens. These neoantigens, derived from somatic mutations, are critical for immune recognition of cancer cells. Consequently, proficient DNA repair may facilitate immune evasion by reducing the visibility of tumors to cytotoxic lymphocytes [128,129].

Conversely, pharmacological inhibition or overload of DNA repair pathways—such as through the use of PARP inhibitors or novel NER-targeting compounds—can promote genomic instability and increase tumor immunogenicity [130]. The accumulation of DNA damage under such conditions leads to the generation of neoantigens, which are presented on the cancer cell surface and can be recognized by T cells, enhancing the anti-tumor immune response [131,132].

In ovarian cancer, particularly among patients harboring BRCA1/2 mutations, inhibition of the BER pathway has been linked to improved outcomes with immunotherapy [133,134]. This effect is partly due to increased neoantigen exposure, which enhances activation of cytotoxic lymphocytes. In such contexts, PARP inhibitors not only induce cancer cell death via synthetic lethality but also boost immunogenicity, making tumor cells more susceptible to immune attack [101,104,112,130]. Combining PARP inhibitors with immune checkpoint blockade (e.g., PD-1/PD-L1 inhibitors) represents a promising therapeutic approach with the potential to significantly improve outcomes in patients with gynecologic malignancies [135,136].

Consequently, the DNA repair mechanisms—BER and NER—play a dual role in tumorigenesis: on one hand, they protect the genome from instability; on the other, they support the survival of cancer cells in an unfavorable microenvironment. Inhibition of these pathways, for example, through the use of PARP inhibitors, may enhance tumor immunogenicity and improve the effectiveness of immunotherapy, particularly in patients with BRCA1/2 mutations.

## 7. Polymorphisms in BER and NER Pathway Genes as Epigenetic Markers

In response to polymorphisms in DNA repair genes of the BER and NER pathways and chronic inflammation, epigenetic changes occur that create a complex epigenetic mechanism [137,138]. These phenomena lead to reduced DNA repair efficiency and accelerate mutagenesis processes and genomic instability [139].

### 7.1. The Role of SNPs in Methylation Processes

DNA repair gene polymorphisms are of significant importance for susceptibility to epigenetic changes, particularly CpG island methylation [140,141]. In the context of gynecological cancers, polymorphisms in the BER and NER pathway genes often lead to reduced efficiency of repair processes [62,63,142]. This is associated with non-synonymous amino acid substitutions that alter protein structures, disrupting their ability to interact with DNA and other proteins in repair complexes. Examples of such variants include rs3212986 (C8092A) in the ERCC1 gene, rs25487 (Arg399Gln) in the XRCC1 gene, rs1052133 (Ser326Cys) in the OGG1 gene, and rs13181 (Lys751Gln) in the XPD (ERCC2) gene, which reduce DNA repair efficiency, leading to the accumulation of DNA damage and potentially inducing an inflammatory state in cells [87,95,143].

Inflammation activates cellular defense mechanisms, including the upregulation of genes encoding DNA methyltransferases (DNMT1, DNMT3A, DNMT3B), which are involved in the regulation of DNA methylation processes [144,145]. Pro-inflammatory cytokines, such as interleukin-6 (IL-6) and tumor necrosis factor-alpha (TNF-α), stimulate signaling cascades that enhance DNMT activity, ultimately leading to the epigenetic silencing of genes involved in DNA repair [146,147]. TNF-α promotes the formation of a signaling complex in which Receptor-Interacting Protein 1 (RIP1) plays a critical role in the activation of Nuclear Factor kappa B (NF-κB), a key transcription factor regulating the expression of DNMT family genes [146,148]. Upon activation, NF-κB triggers the IκB kinase (IKK) complex, which phosphorylates the NF-κB inhibitor IκBα. This phosphorylation event marks IκBα for ubiquitination and subsequent degradation in the proteasome, thereby releasing NF-κB and enabling its translocation into the nucleus, where it can modulate gene expression [149].

**Figure 4:**
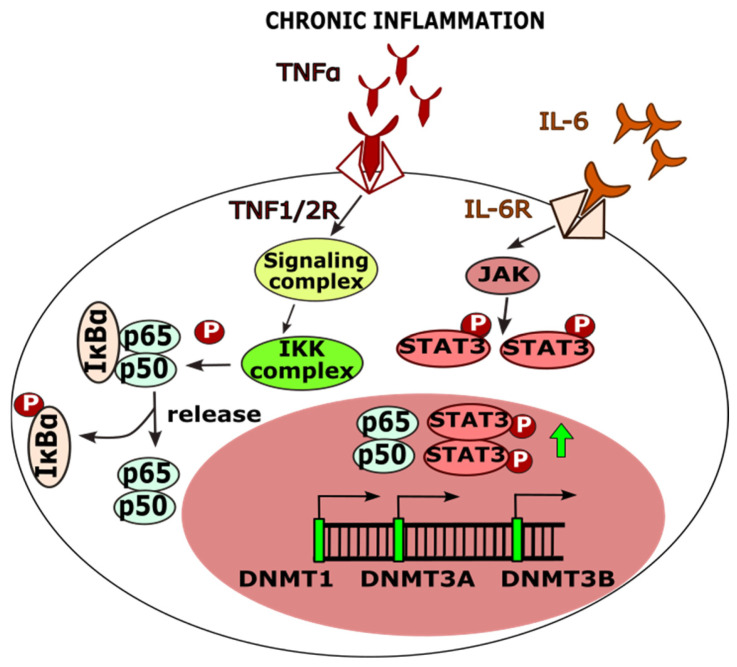
The diagram illustrates the biochemical pathways leading to the induction of gene expression encoding DNMT1, DNMT3A, and DNMT3B proteins (read main text).

In addition, Signal Transducer and Activator of Transcription 3 (STAT3), activated via phosphorylation by Janus kinases (JAK), undergoes dimerization and nuclear translocation, where it regulates the transcription of target genes, including DNMTs [150]. These pathways illustrate how chronic inflammation contributes to an epigenetic environment that represses DNA repair mechanisms (Figure 4).

In gynecological cancers, methylation of CpG islands in the promoter regions of BER and NER pathway genes often coexists with histone modifications that further affect chromatin architecture and gene accessibility [151]. Histone methylation alters chromatin structure, contributing to gene silencing. These epigenetic changes—driven by chronic inflammation and underlying genetic polymorphisms—can promote the accumulation of mutations, genomic instability, and progression of malignancy [23].

### 7.2. Role of SNPs in miRNA-Binding Sites

Beyond epigenetic mechanisms, post-transcriptional regulation mediated by microRNAs (miRNAs) plays a pivotal role in the control of gene expression, including genes involved in DNA repair. This mechanism has been increasingly recognized as a significant contributor to the pathogenesis of gynecological malignancies [152,153,154].

MiRNAs are short non-coding RNA molecules (19–23 nucleotides) that bind to complementary sequences in the 3′ untranslated region (3′UTR) of target mRNAs, leading to translational repression or mRNA degradation [155]. In the context of gynecological cancers, dysregulation of miRNA expression and function impacts DNA damage response pathways, cell proliferation, and apoptosis, thereby contributing to tumor development in ovarian, cervical, and endometrial tissues [152,156].

SNPs located in the 3′UTRs of DNA repair genes—particularly those involved in the BER and NER pathways—can modify miRNA binding sites, interfering with post-transcriptional regulation [157,158]. For example, the C8092A (rs3212986) and T19007C (rs11615) polymorphisms in the 3′UTR of the ERCC1 gene may affect the stability and translation of ERCC1 mRNA by altering miRNA interaction [159]. The C8092A variant may reduce mRNA stability and translation efficiency, impairing DNA repair and potentially promoting tumorigenesis. Although the T19007C variant is synonymous, it may still influence mRNA dynamics and translation, with functional consequences for gene expression regulation [159].

Similarly, the rs6997097 polymorphism in the NEIL2 gene, associated with breast cancer risk, may influence tumor behavior. Patients with the TC genotype at this locus who underwent hormone therapy were reported to have shorter overall and progression-free survival [160].

Alterations in 3′UTR regions that affect miRNA binding can lead to dysregulated gene expression, accumulation of DNA damage, and increased genomic instability. Elucidating the impact of such polymorphisms in DNA repair genes, particularly those within the BER and NER pathways, may provide valuable insights into the molecular mechanisms underlying gynecological cancers and may support the development of novel diagnostic and therapeutic strategies.

## 8. Recommendations

Fully leveraging the potential of SNP-based biomarkers requires further clinical studies aimed at developing strategies to integrate molecular and bioinformatics tools into routine medical practice. Additionally, it is crucial to expand analyses to a polygenic approach that considers interactions between polymorphisms across various DNA repair pathways, enabling the identification of epistatic mechanisms relevant to the risk and progression of gynecological cancers.

The development of a multi-omics approach, integrating genetic data with gene expression, DNA methylation, and proteomic profiles, is essential for assessing the functional consequences of genetic variants. Particular emphasis should also be placed on studying the impact of SNPs on the efficacy of oncological therapies, including platinum-based chemotherapy, radiotherapy, and targeted therapies, using in vitro and in vivo models with defined genetic profiles.

Furthermore, prospective clinical studies on large patient cohorts are necessary to evaluate the influence of SNPs on prognosis and survival. Advanced bioinformatics methods will then enable the construction of predictive models that integrate genetic and clinical data, forming the foundation of personalized medicine.

Additionally, incorporating ethnic diversity in population studies could support the development of individualized diagnostic and therapeutic strategies. Including information on DNA repair gene polymorphisms in prevention and screening programs may improve the effectiveness of personalized oncological surveillance in women at increased risk of developing cancer.

## 9. Conclusions

This review paper highlights the significant role of polymorphisms in DNA repair pathways, particularly BER and NER, as potential biomarkers for assessing the risk and progression of gynecological cancers. It takes into account both molecular determinants and the specific physiology of the female reproductive system, with a focus on translating these findings into clinical applications.

The paper combines an analysis of two key DNA repair mechanisms—BER and NER—and examines their mutual interactions in the context of gynecological carcinogenesis. It not only explores the biological functions of these pathways but also identifies specific SNPs that may serve as markers for the risk of ovarian, endometrial, and cervical cancers. Particular attention is given to their clinical predictive value and potential significance for the development of personalized medicine.

The study evaluates the feasibility of using selected SNPs in diagnostics, disease prognosis, and the personalization of gynecological cancer therapies, including their role in predicting responses to treatments such as platinum-based chemotherapy and PARP inhibitors in the context of specific genetic profiles.

While SNPs in DNA repair genes represent promising biomarkers in gynecological oncology, their clinical application faces significant limitations. Despite an increasing number of studies indicating correlations between specific SNPs and cancer risk, clear and reproducible associations between these variants and clinical features—such as disease stage, treatment response, or prognosis—remain lacking. Additionally, there are practical challenges related to the cost and limited availability of advanced molecular techniques required to identify and validate genetic polymorphisms in clinical settings.

## Figures and Tables

**Table 1:** SNPs in DNA repair pathway genes—BER (data from 2015–2025).

Protein Name	db SNP ID	Number of Cases/Controls	Gynecological Cancer	References
XRCC1	rs1799782	350/400	cervical cancer	[60]
		530,000/260,000	endometrial cancer	[61]
		525/265	cervical cancer	[48]
	rs25489; rs25487	400/400	cervical cancer	[62]
	rs25487	510/510	endometrial cancer	[63]
		213/284	endometrial cancer	[64]
		4028/4953	cervical cancer	[16]
	rs25486	300/300	breast cancer	[65]
XRCC3	rs861539	5740/9931	ovarian, cervical, and endometrial cancers	[66]
APE1	rs1130409	176/177	breast cancer	[59]
SMUG1	rs3087404; rs2029167	400/1200	cervical cancer	[67]
NEIL2	rs804270; rs8191664	400/1200	cervical cancer	[68]
UNG	rs246079	400/1200	cervical cancer	[69]
	rs293795	196/272	ovarian cancer	[70]
OGG1	rs1052133	218/243	endometrial cancer	[71]
		165/200	breast cancer	[72]
		400/400	cervical cancer	[62]
		25,446/41,106	endometrial cancer	[73]
	rs1052133	2712/3638	breast, endometrial and ovarian cancer	[74]
	rs1052133 rs3764959	218/243	endometrial cancer	[71]
	rs293795	196/272	ovarian cancer	[70]
PARP1 LIG3	rs8679 rs4796030	196/272	ovarian cancer	[70]

## Data Availability

No new data were created or analyzed in this study. Data sharing is not applicable to this article.
